# On the Treatment of Pneumonia

**Published:** 1881-02

**Authors:** E W. King


### ON THE TREATMENT OF PNEUMONIA.

By E W. KING, M.D.

I hope I shall be pardoned for presenting the following thoughts upon the plan of treating pneumonia into which I have gradually drifted, and advancing certain views which may be thought by some to be not entirely orthodox. Practitioners of the present have long since realized, what our forefathers did not, that pneumonia is a self-limited disease, and that our duty is not so much combating of the leading symptoms by active and heroic treatment as it is to avert the tendency to death. As has been well said by Jugensen, "Nature cures, and the only duty of the physician is to maintain life until this cure is effected." The writing and teachings of Flint have had much to do in popularizing and impressing the truth upon the minds of the profession in this country; but as public sentiment is prone to oscillate to the opposite extreme from that which it recently occupied, so has been the thought of the medical profession to go to the opposite of the antiphlogistic regimen, and perhaps too much confidence has been reposed in the powers of nature.

Recognizing pneumonia as a self-limited disease, its pathology a croupous inflammation of the air-cells, with an exudation into the air-vesicles and bronchi forming coagulable clots, and that death results, in the majority of fatal cases, as a result of the hyperpyrexia, or from exhaustion and paralysis of the lungs or heart or both, the indications for the management and treatment during the attack are quite clear, viz: to support the powers of life, to combat the hyperpyrexia, and to sustain the action of the respiratory and circulatory systems. The supporting method of treatment in all self-limited diseases is now so generally accepted that we shall not further allude to it. When the accompanying fever runs high, as measured by thermometer, and threatens dissolution from hyperpyrexia, the cold pack, as advised by Niemeyer and practised at Bellevue this winter, or the cold bath of Jurgensen, are certainly advisable if the same results can not be obtained by full doses of quinine.

Jurgensen, Ziemssen's Cyclopedia claims that "death results from insufficiency of the heart." While this is undoubetedly true in many instances, yet from my limited observation, I am led to believe that the greatest danger in uncomplicated croupous pneumonia is in the exhaustion and paralysis of the bronchial tubes in the portions of the lung that are not invaded by the inflammatory action, and that death results more often from paralysis of the lungs from exhaustion than from paralysis of the heart or overwork. In croupous pneumonia the gravity of the case is in proportion to the extent, *cæteris paribus*, of the lung inflamed; not so much from the diffused inflammation as from the inability of the remaining uninflamed lung doing the work for several days that is normally done by the whole respiratory apparatus; and for indications of its failure we look with anxious dread, feeling assured that if we can keep its action free and unembarrassed the disease will soon run its course and recovery ensue. The earliest warnings of this failure are announced to us by the appearance in the healthy lung of rales that partake more of the nature of the sibilant, sonorous, or most usually mucous rales, than of the crepitant or subcrepitant, and indicate a weakness and inability, on the part of the bronchial mucous membrane of that lung, to throw off the accummulated secretion, which prevents the permeation of the air-cells by oxygen for the purification of the blood. To meet this tendency to death, to keep the bronchial tubes free and able to do their work as well as possible under the circumstances, there seems to me that no medicine is more clearly indicated than a stimulating expectorant; and none fulfills that indication better than carbonate of ammonia. Its power to moisten the bronchial mucous membranes and its stimulating qualities have long been recognized, but its applicability to aid the lungs when overworked, as in pneumonia, has not been appreciated as I am convinced it deserves to be. It is in the second stage of the disease that its power is exerted most beneficially, when the inflamed lung is in the stage of red hepatization, when the uninflamed lung is on the verge of exhaustion from prolonged overwork, and the aeration of blood being very imperfect accomplished, then by keeping the fatigued lung stimulated by stimulating expectorants to a normal action we may be able to tide over the crisis successfully, and the consolidated lung terminates in resolution.

It is very probable that much of the advantage to be derived from the administration of carbonate of ammonia, in addition to its stimulating effect, is due to its action in hyperinosis. As Niemeyer has claimed, and which is being generally accepted by the profession as true, the air-vesicles and bronchi are filled with coagulated fibrin, and some claim that the prodomic symptoms which often precede for one or two days the local manifestations should be attributed to the accumulation of fibrin in the blood Others contend that it is not primary but secondary, and is caused and augmented by the local inflamation, and is at its height when the inflamation is the highest. Whichever is true, it is an admitted fact that in pneumonia we have a condition of hyperinosis and a local exudation of fibrin in the air-vesicles of the inflammed lung where it becomes coagulatod indicating quite strongly that excess of fibrin is no insignificant factor in pneumonia.

It is also an admitted fact that no medicines have greater power in liquefying the blood, depriving it of its fibrin more rapidly or promptly, than alkalies; and I think no alkali is better adapted to that duty in croupous pneumonia than the ammonia carbonate, combining, as it does, this with its properties as a stimulating expectorant, making it peculiarly applicable.

Its utility then, from this argument, is in the second stage—the stage of red hepatization—and especially when approaching the stage of resolution, when we wish to deprive the blood and air cells of the excess of fibrin to stimulate the remaining sound lung up to a point of doing its work and maintain as perfect oxygenation of the blood as possible, until resolution has become well established and the crisis passed. With this view I have given it in about sixty cases of pneumonia in the last few years, and now have the gratification of looking back over my notes and finding that not one of those cases under sixty years of age died, and that several who were about that age made good recoveries.

A very common objection to this remedy on the part of the profession is its acrid, pungent, and penetrating smell and taste. This can be obviated and its administration made pleasant by dissolving it in simple syrup, or, as I prefer, in syrup tolu. My usual prescription is—

℞—Carbonate ammonia.......... ʒ j;

Syrup tolu.................... ℥ iss.

M. S. Teaspoonful every half hour or hour, in simple syrup, whisky, or water, as the case demands or patient prefers.

I do not limit myself to the dose of five grains, but have in several cases given it in ten to fifteen-grain doses every half hour for days and nights in succession where there was threatened exhaustion of the uninflamed lung and the case demanded active and continued stimulation to tide over the crisis of resolution. In several cases which presented indications of heart-failure I have deemed it advisable to combine the general stimulant alcohol with ammonia, and the combination makes a very pleasant mixture and reliable stimulant.

Another objection is that ammonia in large doses is an irritant poison; but I assure you if given largely diluted with syrup it is not an irritant to the stomach, and will seldom be considered objectionable to the most fastidious taste.

In cases in which the prognosis is favorable from the very first, and in which I am satisfied active medication will not be necessary, but where we must do something, even if it is a placebo that is prescribed, I usually give this remedy, knowing that its physiological effects are to the contrary of the pathological condition; and by so doing I am intelligently aiding nature to recover her lost equlibrium, and if prompt action should become necessary I will have accomplished something in the right direction and be better enabled to accomplish more.

If my theory is correct that the greater tendency to death in pneumonia is from exhaustion and paralysis of the lung that is not inflamed, I shall most assuredly be justified when I decry the use of opium in any and every form in the second stage of pneumonia. I am well aware that by some of our most prominent teachers and authors opium is considered the sheet-anchor, but I am satisfied in my limited experience that it has never done me well, and where I have been induced to give it because of pain or wakefulness I have had good reason to regret my action. In a few instances, in old persons, where the termination is very doubtful, and I have given it to relieve pain, along with its physiological effects, evidences of failure of the sound lung were manifested, and the patients sank and died. We all know that one of the earliest effects of opium is upon the lungs, producing a slowness of respiration and obtunding the sensibility of the bronchial mucous membranes. Therefure I should unhesitatingly say that it should not be administered in the second stage, especially near its close, for fear of inducing a paralysis or impairing the powers of the lung that must carry on respiration until resolution has taken place.

Since writing the above I have had an opportunity to consult Bartholow's work on The Practice of Medicine, and I find that he strongly recommends the use of ammonia in pneumonia, and prefers the carbonate for its power to lessen the viscidity and coagulation of the fibrinous clots in the air-cells and bronchioles; but from the remark that the "ammonia solution should be continued up to the crisis," implies that he would limit the time of its administration to the early part of the second stage or during the time the exudation of the fibrin is the most active.

It is, however, during the latter part of the second stage, at the approach of and during the crisis, that I have been most gratified with the vigorous administration of ammonia, and especially in adynamic cases; and theoretically, as well as from experience, I still believe I am justified in my conclusions. If the alkali possesses the power to soften or liquefy the fibrinous exudate in the stage when it is being deposited, most assuredly will it have a favorable influence during the stage when nature is exerting herself to accomplish the same object, and especially in cases in which the vitality is so low that some assistance is demanded.

Bartholow's method of administering it is in a solution of liq. ammonia acetatis given every three or four hours. This mixture I do not think is as palatable as that made with syrup; and when we consider the volatile nature of ammonia it is reasonable that its administration should be once in every three or four hours if we desire to maintain its continued effect on the hyperinosis and fibrinous exudate, and derive any benefit from its properties as a stimulating expectorant.—*American Practitioner.*

